# Epitranscriptomics of SARS-CoV-2 Infection

**DOI:** 10.3389/fcell.2022.849298

**Published:** 2022-04-08

**Authors:** Amin Izadpanah, Jay Rappaport, Prasun K. Datta

**Affiliations:** ^1^ Division of Comparative Pathology, Tulane National Primate Center, Covington, LA, United States; ^2^ Department of Microbiology and Immunology, School of Medicine, Tulane University, New Orleans, LA, United States

**Keywords:** epitranscriptome, SARS-CoV-2, RNA modifications, lung, COVID-19

## Abstract

Recent studies on the epitranscriptomic code of SARS-CoV-2 infection have discovered various RNA modifications, such as N6-methyladenosine (m6A), pseudouridine (Ψ), and 2′-O-methylation (Nm). The effects of RNA methylation on SARS-CoV-2 replication and the enzymes involved in this mechanism are emerging. In this review, we summarize the advances in this emerging field and discuss the role of various players such as readers, writers, and erasers in m6A RNA methylation, the role of pseudouridine synthase one and seven in epitranscriptomic modification Ψ, an isomer of uridine, and role of nsp16/nsp10 heterodimer in 2′-O-methylation of the ribose sugar of the first nucleotide of SARS-CoV-2 mRNA. We also discuss RNA expression levels of various enzymes involved in RNA modifications in blood cells of SARS-CoV-2 infected individuals and their impact on host mRNA modification. In conclusion, these observations will facilitate the development of novel strategies and therapeutics for targeting RNA modification of SARS-CoV-2 RNA to control SARS-CoV-2 infection.

## Introduction

The etiological agent of coronavirus disease-2019 (COVID-19) is the novel beta coronavirus, severe acute respiratory syndrome coronavirus-2 (SARS-CoV-2) (Datta et al., 2020; Wu et al., 2020; Zhou et al., 2020). To date, this virus has infected 430 million people and killed more than five million people worldwide (https://coronavirus.jhu.edu/data). The life cycle of SARS-CoV-2 initiates with the viral envelope protein spike (S) binding predominantly the cellular receptor angiotensin-converting enzyme 2 (ACE2) and other potential alternate ACE2 independent receptors, such as CD147, Tyrosine-protein kinase receptor UFO (AXL), Kringle-containing protein marking the eye and the nose protein 1(KREMEN1) and Asialoglycoprotein receptor 1 (ASGR1) (Wang et al., 2020; Wang et al., 2021; Gu et al., 2022). Viral entry via ACE2 dependent mechanism also requires other cellular proteases such as serine proteases (TMPRSS2, TMPRSS11D, and TMPRSS13), furin, and cathepsin L (CTSL) that are involved in the activation of the spike protein through proteolytic cleavage (Bestle et al., 2020; Hashimoto et al., 2021; Hoffmann et al., 2020; Mellott et al., 2021). The release of the viral genome from the endosomal compartment into the cytoplasm is a prerequisite for initiating the viral replication cycle. During the life cycle of SARS-CoV-2, viral proteins and nucleic acid closely interact with many host proteins to regulate viral replication (Gordon et al., 2020; Daniloski et al., 2021; Schmidt et al., 2021). SARS-CoV-2 can alter host nucleic acids and proteins to promote successful viral replication and impair or shut cellular responses to infection (Banerjee et al., 2020).

Many studies have shown that most eukaryotic mRNAs, rRNAs, and tRNAs (Li and Mason, 2014; Roundtree et al., 2017), and viral RNAs (Dang et al., 2019; Tsai and Cullen, 2020; Imam et al., 2020) have multiple forms of RNA modifications that together are defined as the ‘Epitranscriptome’ (Saletore et al., 2012). To date, more than 170 RNA modifications have been identified (Wiener and Schwartz, 2021). N6-methyladenosine (m6A) is the most characterized RNA post-transcriptional modifications (PTM), the other modifications that have been identified are 5-methyl cytosine (m5C), 5-hydroxymethylcytosine (hm^5^C), 7-methylguanosine (m7G), 1-methylguanosine (m1G), pseudouridine (Ψ), N6, N6-dimethyladenosine (m6,2A), ribose-methylation (2′-O-Me), uridine (U), and inosine (I) (Shi et al., 2020). Elucidation of these modifications’ functional roles in RNAs has shown that they play a role in nuclear export of RNAs, pre-splicing of mRNA, stability of RNA, translation initiation, and viral infection (Shi et al., 2020). Concomitantly, several techniques have emerged that enable the identification and study of significant modifications, namely m6A m5C, and Ψ (Sarkar et al., 2021; Wiener and Schwartz, 2021).

### SARS-CoV-2 Transcriptome

**FIGURE 1:**
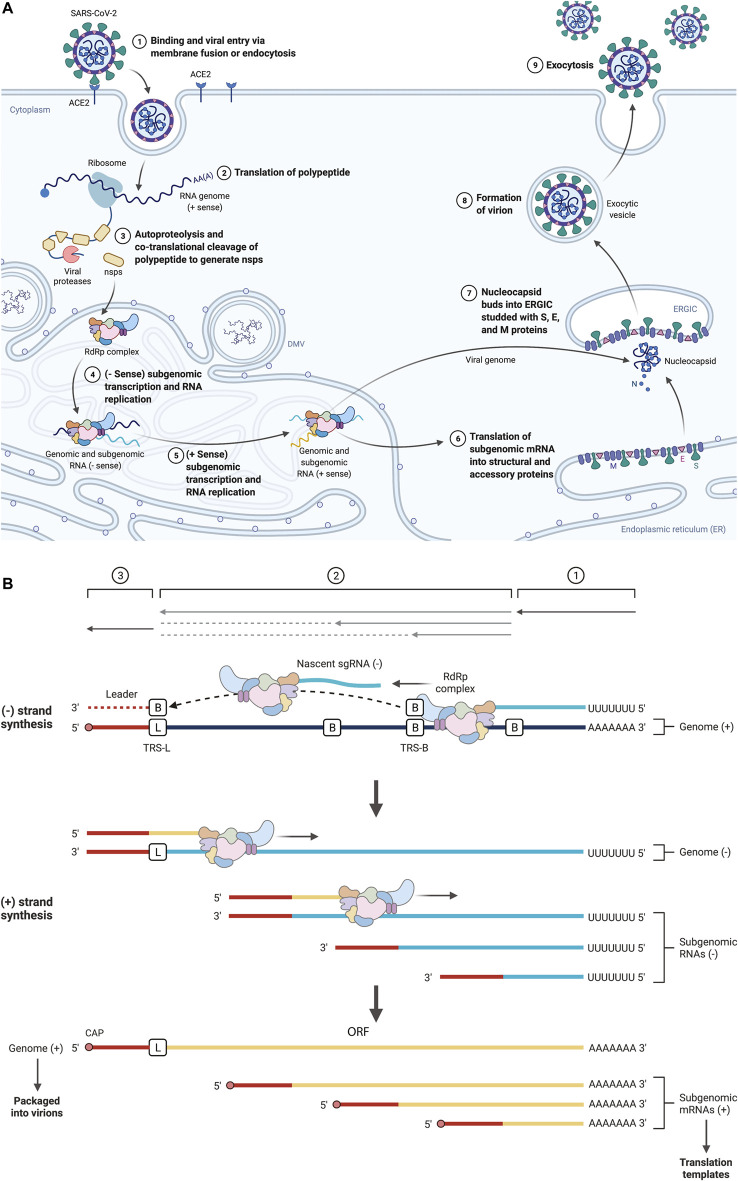
**(A)** Schematic representation of the SARS-CoV-2 biogenesis. SARS-CoV-2 enters host cells interacting with the angiotensin-converting enzyme 2 (ACE2) receptor by the surface spike (S) protein. Upon entry of the virus into the host cell, viral genomic RNA is released into the cytoplasm, where it is translated into viral polymerase proteins. Here, sub-genomic (–) RNAs are synthesized and used as templates for sub-genomic (+) messenger RNAs (mRNAs). The nucleocapsid (N) structural protein and viral RNA are replicated, transcribed, and synthesized in the cytoplasm. In contrast, other viral structural proteins, including the S protein, membrane (M) protein, and envelope (E) protein, are transcribed and then translated in the endoplasmic reticulum (ER). The structural proteins traverse the ER-Golgi intermediate compartment for virion assembly, followed by the release of the nascent virion from the host cell via exocytosis. **(B)** Schematic representation of SARS-CoV-2 sgRNA synthesis. SARS CoV-2 sgRNAs are synthesized via discontinuous transcription. Beginning from the 3′ end of the genomic RNA, RdRp transcribes the body and is halted at the transcription-regulatory sequence in the body (TRS-B). RdRp then resumes transcription at the TRS-L (transcription-regulatory sequence at the leader sequence), a more 5′ location on the genomic RNA. Next, the newly generated negative strand is used as a template for positive-strand synthesis. The TRS is located next to ORFs. Therefore, the positive sense mRNA (sgRNA) produced from the negative-sense RNA contains the leader-sequence fused to the distal ORF. Created with BioRender.

The novel SARS-CoV-2 virus contains a positive-sense, single-stranded RNA genome of ∼30 kb (Wu et al., 2020). The positive sense nature of the genome enables immediate translation, which produces two polypeptides named pp1a (440–500 kDa) or pp1ab (740–810 kDa). The polypeptide pp1a is derived from the open reading frame-1 (ORF1), while the pp1ab is derived from the ORF1ab. ORF1ab occurs due to a -1-ribosome frameshift signal upstream of the ORF1a stop codon, enabling continued translation and thus the larger pp1ab. The polypeptides pp1a and pp1ab are cleaved by viral proteases nsp3 and nsp5 to yield 11 or 15 non-structural proteins (nsps), respectively. Both structural proteins and Nsps depend on translation within the host, but Nsps are viral proteins that are not packaged inside the virus. The nsp12 encodes an RNA-dependent RNA polymerase (RdRp) essential to the coronavirus replication cycle. The RdRp uses the positive-sense genomic template to generate negative-strand RNA. These negative-strand RNA intermediates become the template for producing genomic RNA and positive-sense sub-genomic RNAs (sgRNAs). Genomic RNAs are copies of the original genome of the coronavirus and are thus packaged into the progeny virion (Figure 1A). The sgRNAs contain the same 3′ sequence as the genomic RNA; however, the 5′ ends are different from that of genomic RNA. In coronaviruses, a discontinuous transcription mode occurs during negative-sense RNA synthesis, referred to as leader-body fusion (Sola et al., 2015; Miller and Koev, 2000). Beginning from the 3′ end of the genomic RNA, RdRp transcribes the body and is halted at the transcription-regulatory sequence in the body (TRS-B). RdRp then resumes transcription at the TRS-L (transcription-regulatory sequence at the leader sequence), a more 5′ location on the genomic RNA. The TRS is located next to ORFs. Therefore, the positive sense mRNA (sgRNA) produced from the negative-sense RNA contains the leader-sequence fused to the distal ORF, for example, the S, E, M, and N proteins (Figure 1B). Importantly, sgRNAs encode structural proteins in SARS-CoV-2, including spike protein (S), an envelope protein (E), membrane protein (M), and nucleocapsid protein (N), and accessory proteins 3a, 6, 7a, 7b, 8, and 10.

### Methods for Identification of RNA Modifications in SARS-CoV-2

Several methodologies have been developed to identify RNA modifications (mostly m6A) in eukaryotic cells and viruses. We only describe and compare the techniques used so far to detect SARS-CoV-2 modifications for brevity.

### Antibody-Based Detection Methods

meRIP-seq/m^6^A-seq: (Dominissini et al., 2012; Meyer et al., 2012). In this method, m6A-specific antibodies are used to immunoprecipitate total RNA. Following isolation of m6A bound RNA, the RNA is reverse transcribed to cDNA and deep sequenced using next-generation sequencing (NGS) protocols to obtain high-resolution reads of m6A methylated RNA. The limitations of this technique are 1) issues with m6A antibody specificity, and 2) read lengths are ∼100–200 nucleotides wide, and the bioinformatic prediction of m6A residues is limited to one site per peak.

miCLIP (Linder et al., 2015). In this method, the RNA is first sheared and incubated with anti-m6A antibodies to cross-link RNA using UV light. m6A antibody bound-RNA complexes are recovered by protein A/G-affinity purification, followed by SDS-PAGE and transfer to nitrocellulose membrane. The RNA is then released from the membrane by proteinase K and reverse transcribed to generate a cDNA library using the iCLIP protocol (Hafner et al., 2010). The resulting cDNA is PCR-amplified before sequencing using NGS protocols. The limitations of this technique are 1) issues with m6A antibody specificity, 2) use of excess RNA, and 3) high probability for the introduction of mutation or truncation in relation to the position of the modified adenosine by m6A antibody.

### Liquid chromatography-Tandem Mass Spectrometry (LC-MS/MS)

Oligonucleotide or nucleoside LC/MS (Jora et al., 2019); High-Resolution Ion Mobility Spectrometry-Mass Spectrometry (Kenderdine et al., 2020). In this technique, the total RNA or purified mRNA is digested into individual nucleotides, separated by LC, and quantified by MS. The MS peaks from the sample are compared with the MS peaks of standards to assess all nucleotide modifications in an RNA sample. The limitations of this technique are 1) it requires large amounts of input RNA, 2) no information can be obtained about the location of the modification in an RNA molecule. The advantage of this technique is that even if the levels of the modified nucleotides are low, the measurements are quantitative and reproducible between studies.

### Direct RNA Nanopore Sequencing (DRS)

An alternative to sequencing-by-synthesis is the DRS platform developed by Oxford Nanopore Technologies (Loman et al., 2015). This technique involves ratcheting a single RNA molecule tethered to a motor protein through a protein nanopore sensor (E. coli CsgG-derived nanopore) embedded in a synthetic hydrophobic membrane. The sensor measures changes in an ionic current as RNA passes through the nanopore; information about changes in current and dwell time in the pore identifies the unmodified and modified nucleotide.

Compared to antibody-based methods, the strengths of this technique enable one to detect any RNA modifications in full-length native RNAs at single-nucleotide resolution without the need for reverse transcription or PCR amplification (Depledge and Wilson, 2020; Leger et al., 2021). The limitations are 1) high input RNA required and 2) low throughput.

### RNA Modifications in SARS-CoV-2 RNA

**FIGURE 2:**
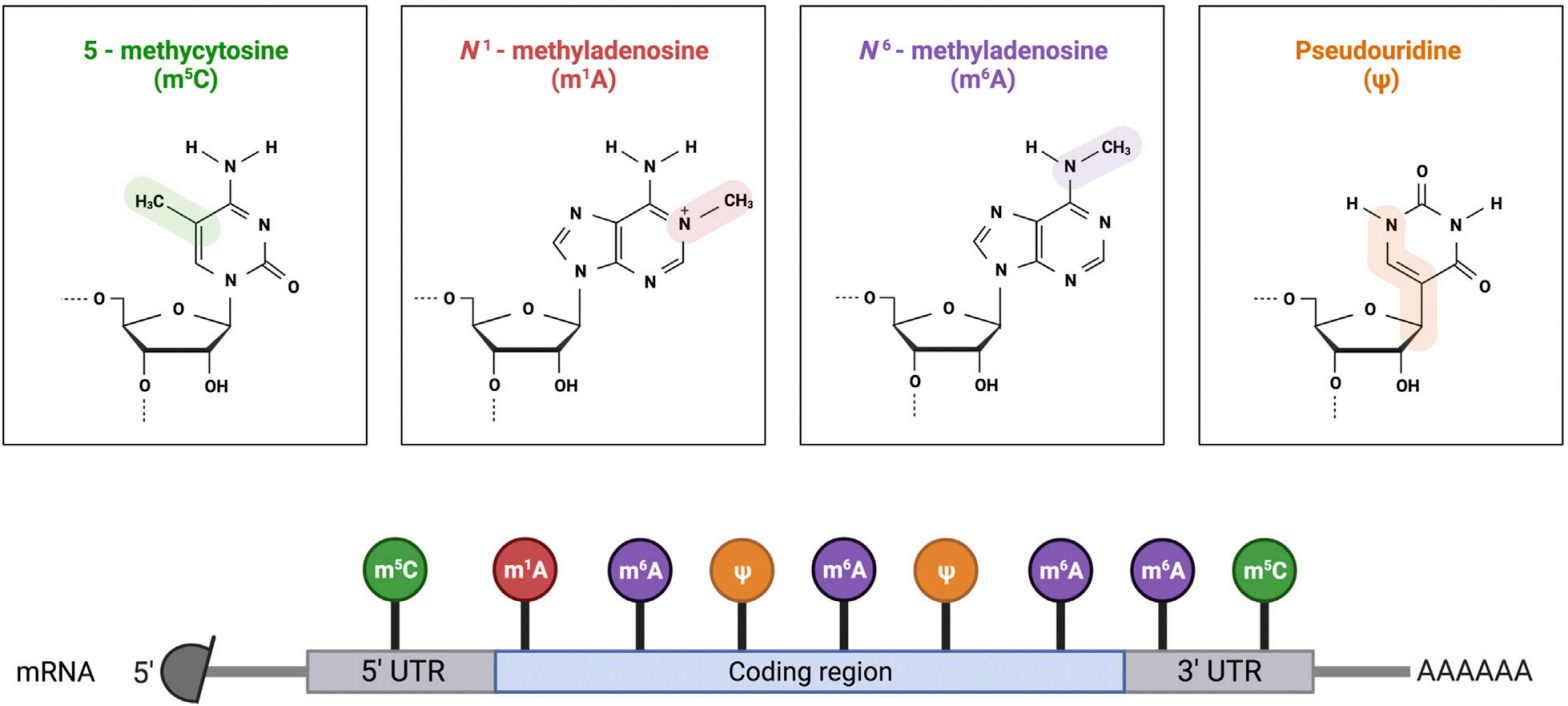
Schematic representation of the common RNA modifications in mRNA. Created with Biorender.

The most predominant RNA modifications are N6-methyladenosine (m6A), N7-methylguanosine (m7G), 5-methylcytosine (m5C), 5-hydroxymethylcytosine (hm5C), pseudouridine (Ψ) and 2ʹ-O-methylations (Nm) (Figure 2). Three different RNA modifications were found in SARS-CoV-2 RNA, namely, m6A, Ψ, and 2′-O-methylation.

The RNA methyltransferase complex is responsible for m6A modification in RNA. This complex is mainly composed of m6A writers METTL3 (methyltransferase like 3), METTL14 (methyltransferase like 14), and WTAP (Wilm’s tumor 1-associated protein). METTL14 forms a heterodimer with METTL3; whereas WTAP is a part of the core complex and influences m6A deposition by METTL3-METTL14 (Little et al., 2000; Bujnicki et al., 2002; Liu et al., 2014; Ping et al., 2014). RBM15 (RNA-binding motif protein 15) plays a role as an adaptor protein essential for the initial recruitment of the writers onto pre-mRNAs (Patil et al., 2016). VIRMA (vir-like m6A methyltransferase associated), an adaptor protein, facilitates interactions with other proteins of the RNA methyltransferase complex (Schwartz et al., 2014). ZC3H13 (Zinc finger CCCH domain-containing protein 13) is a protein that interferes positively with the binding between the adaptor protein RB15 and WTAP (Knuckles et al., 2018).

The addition of Ψ to RNA or pseudouridylation is catalyzed by a class of proteins called pseudouridine synthases (PUS). In humans, there are 13 pseudouridine synthases (Borchardt et al., 2020). Studies have shown that PUS1, PUS7, and TRUB1 pseudouridylate mRNAs (Safra et al., 2017; Carlile et al., 2019).

Viral RNAs predominantly undergo 2′-O-methylation using methyltransferase encoded by the virus itself (Netzband and Pager, 2020).

The first study by Kim and co-workers used the nanopore-based direct RNA sequencing (DRS) approach to study the SARS-CoV-2 transcriptome (Kim et al., 2020). The DRS approach was combined with the complementary DNA nano ball sequencing method to enable a robust investigation of the SARS-CoV-2 transcriptome. Vero cells were infected with SARS-CoV-2 (BetaCoV/Korea/KCDC03/2020). RNA-seq revealed significant read abundance from the 3′ end of the genome, corresponding to the sgRNAs. The high sequencing depth of this method also enabled evaluation of the junctions between the 5′ and 3′ breakpoints, which confirmed the TRS mediated leader-body fusion mechanism of discontinuous transcription for sgRNA synthesis. The transcript frequencies in descending order are N, S, 7a, 3a, 8, M, E, 6, and 7b. Interestingly, Kim et al. identified at least 41 potential RNA modification sites with an AAGAA motif. It was also shown that long viral RNAs (such as gRNA, S, 3a, E, and M) were more frequently modified than the shorter ones (6, 7a, 7b, 8, and N), suggesting that the mechanism of RNA modification may be RNA specific (Kim et al., 2020). Polyadenylation analysis revealed that SARS-CoV-2 sgRNA transcripts have two distinct polyA tail lengths of 30 nucleotides (nt) and 45 nt. These varying lengths may reflect time post-infection. It was shown that polyA tails of bovine coronavirus mRNA increase from 45 nt immediately post-infection to 65 nt 6–9 h post-infection decrease to 30 nt at 120 h post-infection (Wu et al., 2013). Therefore, the authors theorized that the 30 nt polyA tail might reflect aged and decay-prone RNAs (Kim et al., 2020). Forty-one potential sites of base modification (detected by ionic current changes) were correlated with shorter polyA tails, although the type of RNA modification was unknown (Kim et al., 2020).

### N6-Methyladenosine (m6A)

The most common post-transcriptional RNA modification is the N6-methyladenosine (m6A). The m6A modification is commonly found on the DRACH motif (D = A, G, U; R = A, G; H = A, C, U) (Linder et al., 2015). The studies by Liu and co-workers profiled the m6A methylome of SARS-CoV-2 RNA in Vero and Huh7 cells (Liu et al., 2021). To identify the m6A methylome, a refined RNA immunoprecipitation (RIP) strategy was used. Total RNA was subjected to m6A antibody treatment for immunoprecipitation and subsequently sequenced (RIP-seq). In Vero cells, RIP-seq revealed four m6A peaks at 24 h post-infection (hpi) and nine additional m6A peaks at 56hpi. These suggest that m6A modification increases at a later point of infection, supported by increased intensity of m6A at 56 hpi vs. 24 hpi. In Huh7 cells, RIP-seq detected six m6A peaks, corresponding with those in Vero cells at 56 hpi. To determine specific m6A sites, modified m6A individual-nucleotide-resolution crosslinking and miCLIP (Linder et al., 2015) were employed. The studies identified three m6A sites in ORF1ab, one m6A site in ORF 7a, three m6A sites in N, and one m6A site in ORF 10 (Liu et al., 2021). The propensity of the m6A sites to cluster at the 3′ end suggests the involvement of the sgRNAs. Further analysis revealed correlative mutations for each of the m6A sites, except for the second site in ORF1ab. Mutations localizing in the first and third m6A sites (both in ORF1ab) were mainly European strains, while mutations localizing in the fourth and sixth m6A sites (in ORF7a and N, respectively) were primarily in North American isolates. Importantly, m6A sites were also found in the negative-sense strand RNA; however, the ratio of negative-sense to positive sense m6A sites was <1%, and miCLIP could not yield accurate localization. m6A sites on negative-sense RNA suggest a dynamic role for methylation during viral infection and replication.

Using MeRIP-seq to detect m6A modification in the SARS-CoV-2 RNA isolated from infected Vero E6 cells (Zhang et al., 2021), five m6A peaks were identified in the 5′ end (nucleotide 36 to 753 and nucleotide 1,023–1,324) and the 3′ end (nucleotide 27,493 to 27,913, nucleotide 28,475 to 28,706, and nucleotide 28,944–29,751). The m6A residues were in the ORF1ab-, N-, and ORF10-coding regions of the SARS-CoV-2 genome (Zhang et al., 2021). Additional experiments using nanopore-based direct RNA sequencing (DRS) confirmed the specific m6A modification sites in RNAs extracted from Vero E6, A549-ACE2, and Huh7 cells infected with SARS-CoV-2. In brief, six m6A sites were found to be conserved in all the infected cell lines, and the m6A motif in the SARS-CoV-2 genome was mainly GGACA (Zhang et al., 2021). Analysis of SARS-CoV-2 RNA in A549-ACE2 cells and Vero E6 cells revealed nine common m6A sites. However, three m6A peaks in SARS-CoV-2 from Huh7 cells differed from those in Vero E6 and A549-ACE2 cells SARS-CoV-2 RNA. These results show that both conserved and different m6A sites exist in the SARS-CoV-2 genome in other cell lines.

Studies by Campos et al. (Campos et al., 2021) employed DRS to assess the location of m6A residues in SARS-CoV-2 RNA isolated from supernatants of SARS-CoV-2-infected Vero E6 cells that are enriched for genomic RNAs and not sgRNA. They identified fifteen m6A methylated positions, of which six are in ORF N, three in ORF3a, one each in E, M, and ORF7a, and three in ORF7b. In addition, the studies also showed that m6A is associated with the DRACH motif that is highly conserved among variants. However, since the variants Beta (B.1.351) and Eta (B.1.525) have a fourth position C > U change in DRACH at nucleotide position 28,884 of SARS-CoV-2 RNA, this may affect methylation (Campos et al., 2021).

Using MACS2 for peak-calling (Zhang et al., 2008) of meRIP-seq data of SARS-CoV-2 RNA, the studies by Burgess and co-workers (Burgess et al., 2021) identified 14 peak regions in genome length transcripts (gRNA) only, suggesting that both gRNA and sgRNAs harbor m6A residues.

The studies by Li and co-workers (Li et al., 2021) employed liquid chromatography-tandem mass spectrometry (LC-MS/MS and MS/MS/MS) to profile the RNA modifications in SARS-CoV-2 RNA isolated from Vero E6 infected cells. Numerous RNA modifications were detected in SARS-CoV-2 RNA that includes 2′-O-methylated derivatives of all four canonical nucleosides (Am, Cm, Um, and Gm), modified cytidine derivatives (ac4C, m3C, and m5C), two modified uridine derivatives (J and m5U), and two modified adenosines (m6A and m6,6A), and 2-thiocytidine. The authors estimated that m6A accounted for 0.096% of adenosine in the virus and estimated eight m6A-modified sites. The distribution of m6A residues was further validated using MeRIP-seq of full-length SARS-CoV-2 RNA purified from Vero E6 cells. The authors reported that m6A peaks were present in the ORF1ab and 3′ end of the SARS-CoV-2 genome, especially in the N region of SARS-CoV-2 RNA isolated from infected Vero and Caco-2 cells (Li et al., 2021).

Reanalysis of the 41 modified sites reported by Kim and co-workers (Kim et al., 2020) in subsequent studies (Li et al., 2021) found modified adenosine sites embedded in the DRACH motif (D = G/A/U, R = G/A, H = A/U/C) in more than half of the modified sites that were excluded from the analysis of AAGAA motif. This observation revealed the existence of m6A modification in the BetaCoV/Korea/KCDC03/2020 viral genome, which was not reported earlier. The results of the above investigations are summarized in Table 1.

**TABLE 1:** Summary of the studies on SARS-CoV-2 m6A modifications.

Cell line	SARS-CoV-2 Variant	Detection technique	No. of m6A sites or regions	Effect of m6A machinery knockdown (KD) on viral replication (up or down)	References
Vero E6	SARS-CoV-2 (BetaCoV/Korea/KCDC03/2020)	DRS	None	ND	Kim et al. (2020)
Vero E6	SARS-CoV-2 (IVCAS 6.7512)	DRS	14 sites	METTL3 (KD)- Down	Zhang et al. (2021)
				FTO (KD)- Up	
Huh7	SARS-CoV-2 (IVCAS 6.7512)	DRS	9 sites	ND	Zhang et al. (2021)
A549/ACE2	SARS-CoV-2 (IVCAS 6.7512)	DRS	9 sites	ND	Zhang et al. (2021)
Caco-2	SARS-COV-2 (USA-WA1/2020)	MeRIP-seq	13 regions*	METTL3 (KD)- Down	Li et al. (2021)
Vero	SARS-COV-2 (USA-WA1/2020)	MeRIP-seq LC/MS-MS/MS	27 regions*5 regions# 8 sites	ND	Li et al. (2021)
Huh7	SARS-CoV-2 (BetaCov/Wuhan/IME-BJ01/2020)	MeRIP-seq	7 regions	METTL3 (KD)- Up METTL14 (KD)- Up ALKBH5 (KD)- Down YTHDF2 (KD) -Up	Liu et al. (2021)
Vero E6	SARS-CoV-2 (BetaCov/Wuhan/IME-BJ01/2020)	MeRIP-seq miCLIP	4 regions (24 h) 13 regions (56 h) 8 sites (56 h)	ND	Liu et al. (2021)
Vero E6	SARS-CoV-2 (Brazil)	DRS	15 sites	ND	Campos et al. (2021) Burgess et al. (2021)
A549/ACE2	SARS-COV-2 (USA-WA1/2020)	MeRIP-seq DRS	14 regions 1 site	METTL3 (KD)- Down YTHDF1 (KD)-Down YTHDF3 (KD)-Down	

*All reads. #, no duplicates. ND, not determined.

### Pseudouridine (Ψ)

Pseudouridine (Ψ), residue formed by isomerization of uridine (U), is found at high levels (>1%) in eukaryotic rRNA and tRNA and at lower levels (<1%) in eukaryotic mRNA (Carlile et al., 2014; Borchardt et al., 2020). Ψ residues are also seen in viral RNAs (McIntyre et al., 2018; Furuse, 2021). Using DRS, five high confidence Ψ sites in TRS-S, five in TRS-3a, five in TRS-E, and five in TRS-M of SARS-CoV-2 sgRNAs were detected (Fleming et al., 2021). In TRS-3a, -E, and -M, five identified peaks mapped to the nucleotide (U27164, U28039, U28759, U28927, and U29418). Biochemical validation showed that recombinant PUS1 and PUS7 introduced Ψ in synthetic RNAs at positions U28927 and U29418, while recombinant PUS1 introduced Ψ in synthetic RNAs at position U29418 (Fleming et al., 2021).

### 2′-O-Methylation

Numerous studies on RNA capping in coronaviruses showed the involvement of several non-structural proteins (nsps): nsp13, a bifunctional helicase and RNA/NTP triphosphatase; nsp14, a bifunctional mRNA cap guanine-N7 methyltransferase and 3′→5′ mismatch exonuclease; nsp16, a cap ribose 2′-O methyltransferase; and a guanylyltransferase (Ivanov and Ziebuhr, 2004; Bouvet et al., 2010; Chen et al., 2011; Bouvet et al., 2012). 2′-O methylation occurs on the ribose sugar of the first nucleotide of SARS-CoV-2 mRNA. It is catalyzed by a complex of SARS-CoV-2 nsp16 and nsp10 in the presence of cognate RNA substrate analog and methyl donor, S-adenosyl methionine (Viswanathan et al., 2020). In addition, the high-resolution structure of the ternary complex of the SARS-CoV-2 RNA cap/nsp16/nsp10 complex showed that the ligand-binding site in nsp16/10 allows accommodation of small molecules outside of the catalytic pocket (Viswanathan et al., 2020). In a recent study using Nm-seq, a total of 130 2′-O methylation sites were identified in the SARS-CoV-2 genome. The 2′-O methylation was enriched in the 5′ and 3′ UTRs of SARS-CoV-2 (Yang et al., 2021).

### m6A Modification in SARS-CoV-2: Role of Writers, Erasers, and Readers

The writer enzymes add the m6A modification, recognized by the reader enzymes and removed by the eraser enzymes. Readers play a critical role in identifying the m6A mark and regulating the fate of m6A-marked mRNA; for brevity, we only discuss the readers, writers, and erasers that play a role in SARS-CoV-2 RNA modifications. Characterization of the writer protein complex involved in m6A modification in mRNA is methyl-transferase-like three METTL3 and its homolog METTL14 (Bujnicki et al., 2002; Liu et al., 2014). Both proteins act synergistically by forming a stable heterodimer METTL3 and METTL14 (1:1 stoichiometry) to facilitate m6A addition to mRNA methylation in cells (Liu et al., 2014). More recently, a third critical component of this complex, Wilm’s tumor-associated protein (WTAP), which lacks methyltransferase activity, was essential for the methylation process (Little et al., 2000; Ping et al., 2014). WTAP interacts with the METTL3/METTL14 heterodimer to direct the localization of this m6A writer complex to nuclear speckles, where splicing occurs (Liu et al., 2014). Interestingly, RNA-binding proteins, RNA-binding motif protein 15 (RBM15), and its paralogue RBM15B were shown to direct the writer complex to the XIST long non-coding RNA and several cellular mRNAs to guide site-specific methylation of target RNA (Patil et al., 2016). Zinc finger CCCH domain-containing protein 13 (ZC3H13) was identified as a nuclear m6A writer gene (Wen et al., 2018).

Among the m6A erasers, the first protein identified was fat mass and obesity protein (FTO), an alpha-ketoglutarate-dependent dioxygenase member of the AlkB family that demethylates RNA (Jia et al., 2011). AlkB homolog 5, RNA demethylase (ALKBH5), another member of the AlkB family, also promotes mRNA demethylation rates similar to FTO (Zheng et al., 2013).

The cytosolic m6A readers are the YT521-B homology domain-containing proteins (YTHDF1-3) that regulate target mRNA fate. YTHDF1-3 have a highly conserved single-stranded RNA-binding domain at the carboxy terminus (the YTH domain) and a less conserved amino-terminal region (Zhang et al., 2010). YTHDF1 enhances ribosome assembly of m6A mRNA and interacts with the translation initiation factor 3 (eIF3) to promote translation of m6A containing mRNA (Wang et al., 2015). YTHDF2 promotes the degradation of non-translating m6A-modified mRNAs (Wang X et al., 2014). YTHDF3 promotes the translation and degradation of mRNA (Shi et al., 2017). YTH domain-containing 1 (YTHDC1) is a nuclear m6A reader protein that mediates mRNA metabolism and regulates mRNA splicing (Xiao et al., 2016). YTH domain-containing 2 (YTHDC2) is an m6A reader protein with a 3′-5′ RNA helicase activity, binds to m6A mRNA, and regulates mRNA translation and stability (Wojtas et al., 2017). Insulin-like growth factor-2 (IGF2) mRNA-binding proteins 1, 2, and 3 (IGF2BP1/2/3) are a new family of m6A readers that prevents decay of m6A-modified mRNAs (Nielsen et al., 1999; Bell et al., 2013; Huang et al., 2018).

To explain the role of writers, erasers, and readers in the SARS-CoV-2 biogenesis, the studies by Zhang and co-workers (Zhang et al., 2021) showed that METTL3 overexpression in Vero E6 cells induced an abundance of m6A containing viral RNA levels, while METLL3 or FTO knockdown significantly decreased or increased virus levels, respectively. This study also showed that SARS-CoV-2 infection induced the expression of METTL3 and altered distribution in both the nucleus and cytoplasm and co-localized with RdRp. In addition, METTL14, WTAP, ALKBH5, and FTO were shown to co-localize with the viral protein N. These observations taken together suggest that SARS-CoV-2 infection affects host cells m6A methyltransferase and demethylases.

Using A549 cells expressing human ACE2 receptor and a SARS-CoV-2 reporter virus expressing mNeonGreen (icSARS-CoV-2-mNG) as a model of SARS-CoV-2 infection, the effects of knockdown of METTL3, YTHDF1, YTHDF2, and YTHDF3 was used tested (Burgess et al., 2021). The studies showed that siRNAs against METTL3 reduced the percentage of infected cells by 78–81% compared to control cells. While, (knock-down of YTHDF2 or YTHDF3 reduced the percentage of infected cells by 42–66% or 75–76% of the control, respectively. In contrast, the knockdown of YTHDF1 reduced SARS-CoV-2 infection by 23–89%, dependent upon the targeting siRNAs used. Co-depleting all three YTHDF1, YTHDF2, and YTHDF3 reduced the number of infected cells by 72% (Burgess et al., 2021).

**FIGURE 3:**
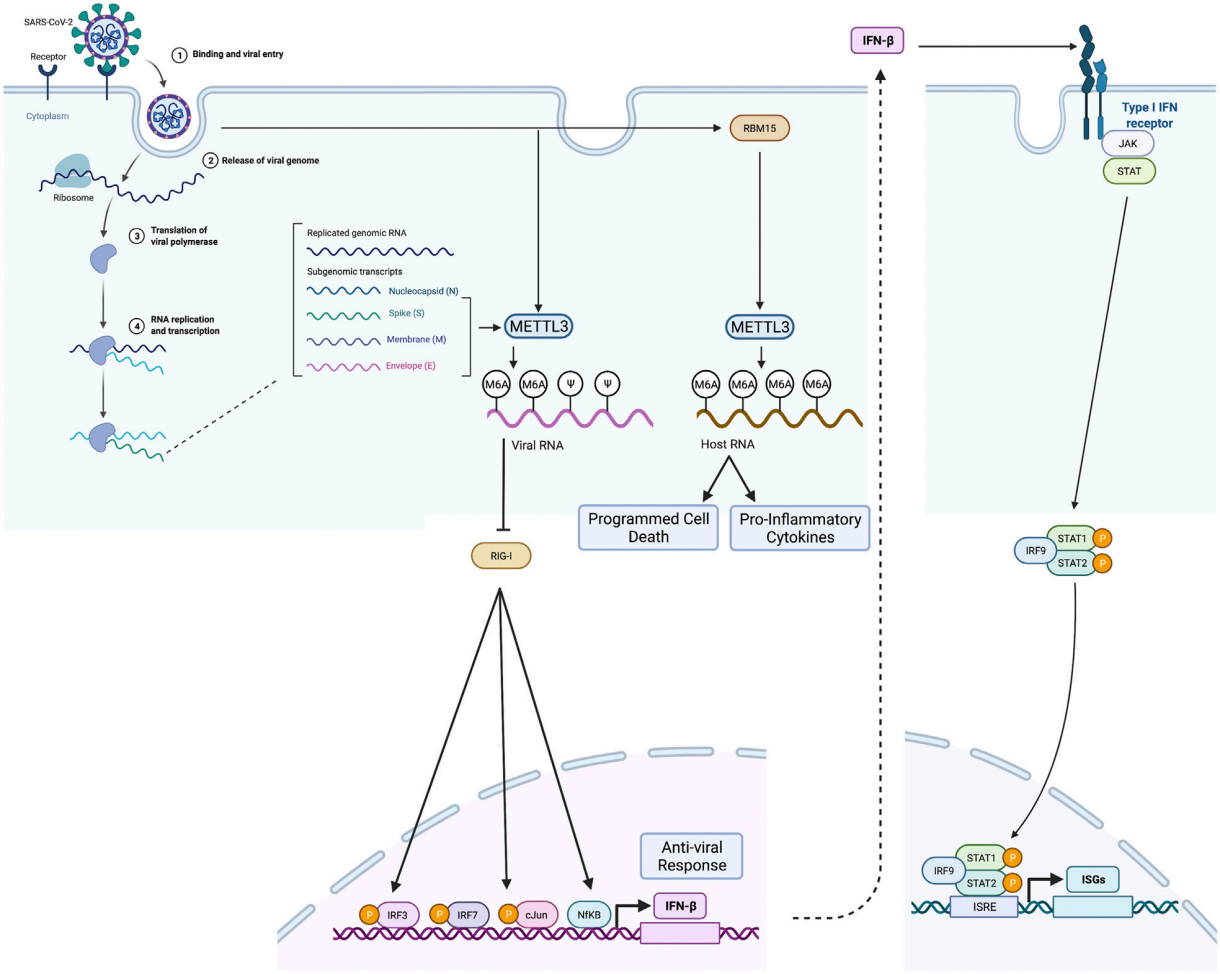
m6A modification in SARS-CoV-2 RNA and cellular mRNA by METLL3 and RBM15, respectively, and its effect on evasion of host innate immune response by SARS-CoV-2 and host cell. (Left) After entering the host cell, the viral genome enters the replication phase, and METTL3 introduces m6A residues in viral RNA. m6A methylated SARS-CoV-2 does not bind RIG-I and induces the expression of antiviral molecules such as IFNβ. Viral infection also enhances METTL3 and RBM15, leading to enhanced host mRNA m6A methylation resulting in activation of inflammatory gene expression and programmed cell death. (Right) When antiviral molecules such as IFNβ levels increase, it leads to activation JAK-STAT pathway and interferon-stimulated gene (ISG) expression. Created with BioRender.

Liu and co-workers further investigated the role of METTL3, METTL14, ALKBH5, and YTHDF2 by generating individual knockdown cell lines (Liu et al., 2021). Knockdown of METTL3, METTL14, and YTHDF2 significantly increased viral infection and replication, while knockdown of ALKBH5 decreased viral infection and replication. YTHDF2 was shown to induce the decay of m6A transcripts. Therefore, the authors concluded that host m6A methylome regulators play a critical role in SARS-CoV-2 replication. In the studies by Li and co-workers (Li et al., 2021), knockdown of METTL3 using two different small hairpin RNAs in Caco-2 cells infected with SARS-CoV-2 resulted in a reduction of SARS-CoV-2 viral load and proviral gene expression. MeRIP-seq analysis of SARS-CoV-2 isolated from METTL3 knock-down (METTL3-KD) Caco-2 cells showed decreased m6A occupancy in the N region of the SARS-CoV-2 virus (Li et al., 2021). Li et al. also demonstrate that the presence of m6A on the SARS-CoV-2 RNA avoids sensing by RIG-1 (retinoic acid-inducible gene 1) (Li et al., 2021) (Figure 3). RIG-1 is a cytosolic pattern recognition receptor essential to innate immunity through its ability to activate the type 1 interferon response (Figure 3).

Collectively, these studies demonstrate that a functioning m6A RNA modification pathway is beneficial to SARS-CoV-2 replication. Further, it provides proof of concept that targeting cellular components of this intensively studied RNA modification pathway could ultimately lead to new therapeutic opportunities to control these important viral pathogens.

### Impact of SARS-CoV-2 on Host Cell m6A Methylome

Many studies have documented that RNA modification of viral RNAs through methylation plays a role in the evasion of the host’s innate immune response (Kariko et al., 2005; Durbin et al., 2016; Lu et al., 2020). Conversely, viral infection may also play a role in regulating host methylation. Indeed, in recent studies, SARS-CoV-2 infection influenced the host cell m6A methylome. RNA seq analysis of METTL3-KD SARS-CoV-2 infected Caco-2 cells showed differential expression of genes involved in regulating metabolic processes, immune response, RNA processing, ribosome function, protein modification, DNA repair, cell cycle, and cell differentiation (Li et al., 2021). However, knockdown of METTL3 in Calu-3 cells infected with SARS-CoV-2 significantly enhanced IFNβ and ISG expression (STAT1, STAT2, and IRF7) and cytokine/chemokine expression (IL-6, IL-8, TNF, CXCL10/IP10, CCL5, CXCL1, CXCL3, and CCL20), while METTL14 KD only increased IFNγ, IL-6, TNF, and CXCL3 levels (Li et al., 2021).

m6A epitranscriptomic microarray studies using peripheral blood samples of COVID-19 patients and healthy controls showed altered m6A modification levels in lymphocytes (Meng et al., 2021). Analysis of m6A regulators identified RBM15, WTAP, YTHD3 and IGF2BP1 overexpression and down regulation of METTL16, YTHDF2, YTHDC2 and IGF2BP2 in patients with COVID-19. Hypermethylation of caspase (CASP) 1, CASP5, and tribbles homolog one gene (TRIB1), thymic stromal lymphopoietin (TSLP), DEAD-box helicase 3 X-linked (DDX3X), and interleukin 17 receptor B (IL17RB) mRNAs and subsequent translation promotes programmed cell death and activation of an abnormal inflammatory response in severe patients (Meng et al., 2021).

### Pseudouridine (Ψ) in SARS-CoV-2 Vaccines

Epitranscriptomic modifications may be leveraged for therapeutic mRNA vaccine design. Indeed, both Pfizer and Moderna mRNA vaccines utilize Ψ. The Pfizer-BioNTech vaccine (BNT162b2; trade name: Comirnaty; generic name: tozinameran) is an mRNA-based vaccine, whereby synthetic mRNA coding for the SARS-CoV-2 spike protein is encapsulated within a lipid nanoparticle. Upon intramuscular administration, the lipid nanoparticle facilitates delivery of the mRNA payload into local muscle cells or infiltrating immune cells. The mRNA is then translated to produce spike protein, which may be delivered to the plasma membrane as an anchored spike protein or processed and presented by the major histocompatibility complex (MHC), thus generating an immune response. Despite inducing a protective immune response, mRNA vaccine technology has a significant downside to forming undesirable RNA structures. This may cause uncontrolled immune activation, which is detrimental to the host and may also decrease protein translation by inhibiting the ribosome and activating mRNA-degrading ribonucleases, which impairs the efficacy of the mRNA vaccine. The beneficial effects of incorporating Ψ in mRNA vaccines maybe because of the reduction of undesirable RNA structures (Nance and Meier, 2021).

Ψ may reduce the synthesis of antisense RNA, thus improving the translational efficiency of mRNA vaccines1. Ψ may also reduce the formation of double-stranded RNA (dsRNA) (Nance and Meier, 2021). dsRNA is recognized by TLR3 and RIG-1 and initiates the expression of inflammatory genes through NF-κB and IRF activities (Kariko et al., 2004; Durbin et al., 2019; Mauger et al., 2019). Single-stranded poly-uridine has been shown to induce TLR7 activation, an effect that is reduced with Ψ because of the steric incompatibility (Heil et al., 2004; Mauger et al., 2019). N1-methyl-Ψ is the form used in the mRNA vaccines against SARS-CoV-2 (Morais et al., 2021). The methylated-N1 prevents irregular bonding with guanine, uracil, or cytosine and promotes faithful bonding of N1-methylated Ψ with adenine. Consistently, N1-methyl-Ψ inhibited activation of innate immune sensors such as TLR and showed improved protein translation *in vivo* (Andries et al., 2015).

Altogether, while not explicitly demonstrated robustly in current COVID-19 vaccines yet, these favorable properties of Ψ, which reduce unwanted immunostimulatory potential, justify its adoption in current mRNA vaccines and perhaps in future mRNA-based therapeutics. However, it is essential to note that the location of Ψ within the transcript can influence the net effect on translation. For example, Ψ within the 5’ UTR may block translation initiation, while Ψ within the coding sequence may increase the functional half-life of the mRNA and thus increase translation. Further, once the mRNA of the vaccines is degraded, it is unknown how the modified nucleosides may affect cellular physiology. Characterization of the breakdown and potential salvaging of modified nucleotides from vaccine mRNA is an area that requires further study and attention.

Exploiting base modifications in mRNA vaccines is critical to improving efficacy. This is exemplified by the Curevac COVID-19 mRNA vaccines (CVnCoV), which utilized unmodified mRNA, yielded only 48% efficacy against disease severity (Kremsner et al., 2021). And while these suboptimal results, compared to Pfizer and Moderna vaccines with ∼90% efficacy, maybe due to the lower dose used, it is still likely that using N1-methyl- Ψ mRNA would have increased effectiveness (Dolgin, 2021; Morais et al., 2021).

## Conclusion and future Directions

In summary, RNA modifications and the enzymatic machinery are emerging as essential players in regulating SARS-CoV-2 mediated infection and subsequent COVID-19 disease progression. m6A modifications are differentially expressed in host mRNAs in COVID-19 patients, and some essential RNA modifications such as m6A, Ψ, and 2′-O-methylation in SARS-CoV-2 RNA have been identified that permits the virus to interfere with the host’s natural immune response and thereby create a haven for viral replication specifically in the lungs in infected individuals. In this regard, studies in diverse viral infections showed that m6A and 2′-O-methylation control antiviral type I IFN (Kong et al., 2019; Winkler et al., 2019) and interferon gene (ISG) responses (Zheng et al., 2017; Williams et al., 2020).

Future studies at the tissue level are needed to identify the actual writer, reader, and eraser enzyme (s) and PUS synthases in the course of SARS-CoV-2 infection in organs other than the lung. Studies are also needed to determine if pseudouridylation and 2′-O-methylation of SARS-CoV-2 RNA allow the virus to escape host immune response.

There are a plethora of vaccines available to prevent infection (Tatsi et al., 2021) and clinical treatment that includes potential antiviral drug candidates, monoclonal antibodies, and glucocorticoids (Gavriatopoulou et al., 2021) to mitigate the severity of COVID-19. The therapeutic modulation of RNA modification enzymes that are altered or dysregulated during SARS-CoV-2 infection could be a new strategy for attenuating SARS-CoV-2 replication. The strategy that can be employed is to use inhibitors or activators of METTL3, METTL14, YTHDF2, ALKBH5, and FTO to control SARS-CoV-2 replication by modulating the m6A methylome. In this context, selective inhibition of METTL3 catalytic activity using STM2457 inhibits HCoV-OC43 and SARS-CoV-2 infection (Burgess et al., 2021). Since YTHDFs are involved in orchestrating innate antiviral immunity, we can consider them as potential targets of viral countermeasures. Since the ligand-binding site in SARS-CoV-2 nsp16/10 can accommodate small molecules outside of the catalytic pocket, besides SAM- and RNA cap-binding pockets, it is prudent to screen for inhibitors as antiviral therapies to treat SARS-CoV-2 infections.
